# Identification of a Novel Ciprofloxacin Tolerance Gene, *aciT*, Which Contributes to Filamentation in Acinetobacter baumannii

**DOI:** 10.1128/AAC.01400-20

**Published:** 2021-05-18

**Authors:** Varsha Naidu, Bhumika Shah, Karthik S. Kamath, Arthur Chien, Stephanie Nagy, Alaska Pokhrel, Mark Molloy, Karl A. Hassan, Ian T. Paulsen

**Affiliations:** a Department of Molecular Sciences, Faculty of Science and Engineering, Macquarie University, Sydney, NSW, Australia; b Australian Proteome Analysis Facility (APAF), Macquarie University, Sydney, NSW, Australia; c The Microscopy Unit, Faculty of Science and Engineering, Macquarie University, Sydney, NSW, Australia; d Faculty of Medicine and Health, University of Sydney, Sydney, NSW, Australia; e School of Environmental and Life Sciences, University of Newcastle, Newcastle, NSW, Australia

**Keywords:** *Acinetobacter*, fluoroquinolone, antibiotic resistance, ciprofloxacin, filamentation, pathogen

## Abstract

Fluoroquinolones are one of the most prescribed broad-spectrum antibiotics. However, their effectiveness is being compromised by high rates of resistance in clinically important organisms, including Acinetobacter baumannii. We sought to investigate the transcriptomic and proteomic responses of the clinical A. baumannii strain AB5075-UW upon exposure to subinhibitory concentrations of ciprofloxacin. Our transcriptomics and proteomics analyses found that the most highly expressed genes and proteins were components of the intact prophage *phiOXA.* The next most highly expressed gene (and its protein product) under ciprofloxacin stress was a hypothetical gene, ABUW_0098, named here the Acinetobacter ciprofloxacin tolerance (*aciT*) gene. Disruption of this gene resulted in higher susceptibility to ciprofloxacin, and complementation of the mutant with a cloned *aciT* gene restored ciprofloxacin tolerance to parental strain levels. Microscopy studies revealed that *aciT* is essential for filamentation during ciprofloxacin stress in A. baumannii. Sequence analysis of *aciT* indicates the encoded protein is likely to be localized to the cell membrane. Orthologs of *aciT* are found widely in the genomes of species from the *Moraxellaceae* family and are well conserved in Acinetobacter species, suggesting an important role. With these findings taken together, this study has identified a new gene conferring tolerance to ciprofloxacin, likely by enabling filamentation in response to the antibiotic.

## INTRODUCTION

Acinetobacter baumannii is a Gram-negative bacterium that is considered a serious threat to global health care due to increasing rates of multidrug resistance (MDR) in clinical isolates. MDR clinical isolates of A. baumannii can be a challenge to treat for clinicians as few therapeutic options are available. The World Health Organization (WHO) has classified A. baumannii as one of the top three priority pathogens for which new antibiotic development is needed urgently. Broad-spectrum antibiotics, including fluoroquinolones such as ciprofloxacin, have been extensively used to treat infections caused by A. baumannii. Ciprofloxacin is listed as an essential medicine by the WHO and has been reported by the CDC to be one of the most prescribed oral antibiotics. Surveillance studies have shown significant increases in resistance rates for ciprofloxacin in A. baumannii, thereby compromising its use in health care. Discovery and characterization of novel ciprofloxacin-resistant genes are therefore critical for future inhibitory drug designs.

Fluoroquinolones exert their antibacterial activity by interfering with the function of type IIA topoisomerases—DNA gyrase and topoisomerase IV (1). Type IIA topoisomerases modulate the topological state of DNA through negative supercoiling of DNA and by decatenating newly replicated DNA during transcription and replication. Quinolones form a stable ternary complex with topoisomerase and DNA, preventing DNA strands from religating, resulting in double-stranded DNA breaks (2).

In the well-studied Escherichia coli model, stalling of replication forks leads to RecA-mediated autoproteolytic cleavage of the repressor LexA (3), which eventually self-cleaves, leading to the derepression of more than 30 genes associated with DNA repair and error-prone DNA polymerases (4). Collectively this process is referred to as the SOS DNA damage response (5). The cell division inhibitor SulA which is part of the SOS response, temporarily arrests cell division, allowing time for DNA repair to occur (6). During this process, bacterial cells change shape to form long filamentous structures. Filamentation has been described to occur in response to stressful environments, including DNA damage, antibiotics (7), and desiccation (8), and to subvert host innate defenses (9). In E. coli, filamentation in response to sub-MICs of ciprofloxacin has been shown to generate multiple mutant chromosomes that asymmetrically divide at the filament tips, giving rise to resistant offspring cells (10): hence, filamentation is a significant precursor in the evolution of resistance. Homologs of LexA and SulA, as well as several other DNA damage and cell division genes, have not been identified in Acinetobacter species (11, 12). It is unclear how Acinetobacter responds to genotoxic antibiotic ciprofloxacin at the core genome level.

In A. baumannii, mutations that lead to ciprofloxacin resistance tend to primarily emerge at the Ser81 codon of the DNA gyrase subunit GyrA, followed by secondary mutations in the Ser84 codon of the topoisomerase IV subunit ParC (13–16). Collectively, codon substitutions at these locations in the *gyrA* and *parC* genes have been shown to increase resistance to the fluoroquinolone ciprofloxacin by ∼128-fold (17). In our current study, the global response to ciprofloxacin was investigated using transcriptomics and proteomics in the ciprofloxacin-resistant A. baumannii isolate AB5075-UW, which carries a mutation at the Ser81 codon of GyrA. This enabled the identification of genes in the core A. baumannii genome that are involved in tolerance to ciprofloxacin.

## RESULTS AND DISCUSSION

### Global transcriptomic and proteomic response to ciprofloxacin.

**FIG 1:**
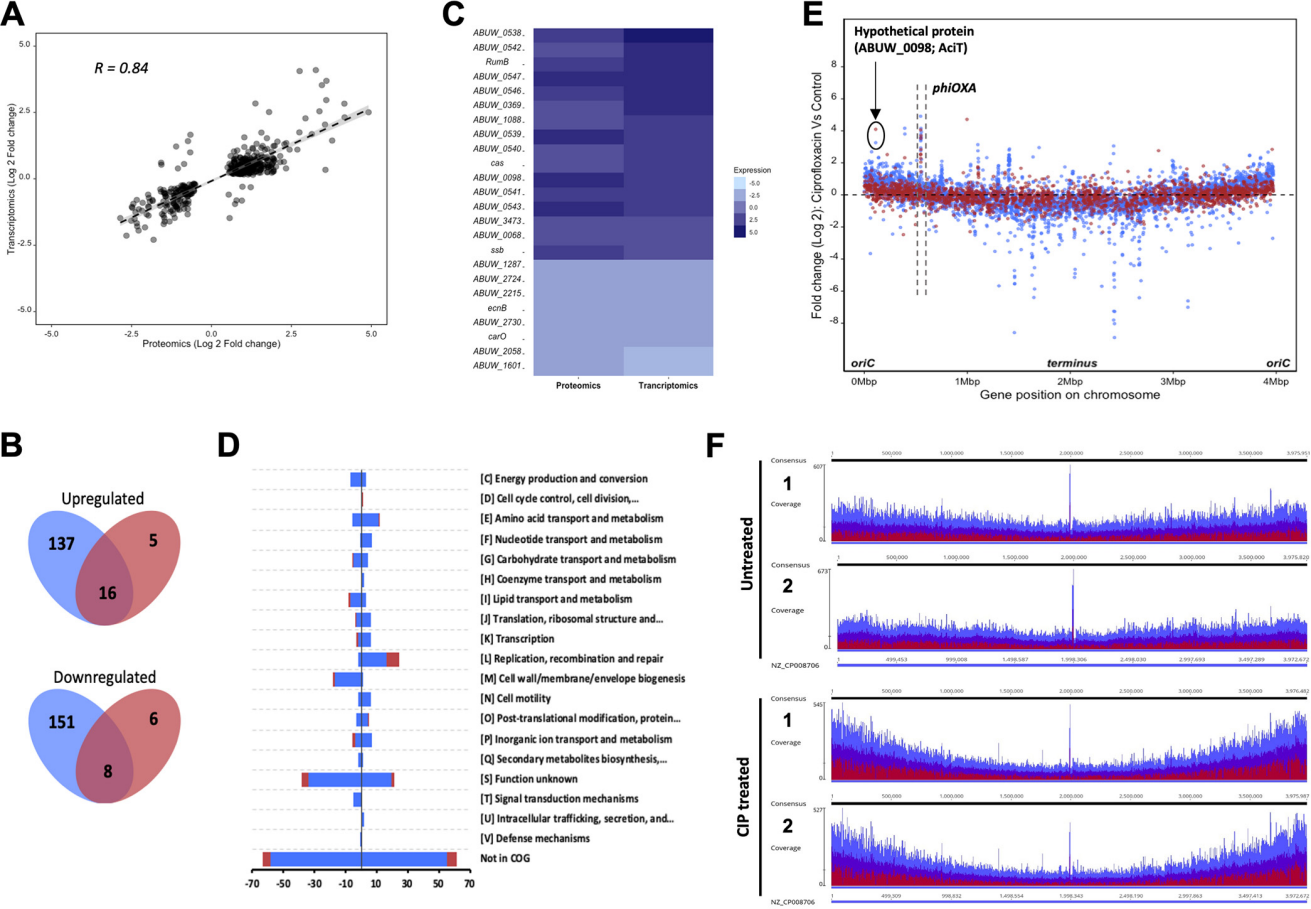
(A) Correlation between transcriptomics and proteomics data after they were filtered based on an adjusted *P* value of 0.05. The Pearson correlation coefficient between both data sets is 0.84. (B) Venn diagram depicting the relationship between genes and proteins that had a log_2_ fold change of greater or less than 1.5 (adjusted *P* value of <0.05). The proteomics data set is represented by the blue oval, and the transcriptomics data set is represented by red. The expression of 16 intersecting genes and proteins was greater than 1.5 (log_2_ fold change), and the expression of 8 was less than 1.5 (log_2_ fold change). (C) Intersecting genes and proteins from the Venn diagram were identified and plotted in a heat map. (D) Functional class analysis of genes/proteins based on COG categories. Bars represent the number of genes (blue) or proteins (red) in each COG category that had either increased or decreased expression by a log_2_ fold change of greater than 1.5 or less than 1.5 (adjusted *P* value of <0.05), respectively. (E) The transcriptional (blue) and proteomic (red) response of A. baumannii AB5075-UW to ciprofloxacin shock treatment. Each point in the graph represents a single ORF within the genome, arranged according to their location in the genome on the *x* axis and their fold change (log_2_) in expression on the *y* axis, following treatment with 31.25 μg/ml ciprofloxacin for 1 h. (F) Distribution of DNA reads along AB5075-UW chromosome of ciprofloxacin-treated (31.25 μg/ml, 1 h) and control (no treatment). The height of the graph shows the coverage of reads represented as minimum (red), average (purple), and maximum (blue).

An international clonal complex I A. baumannii strain AB5075-UW (18), with a ciprofloxacin MIC of 125 μg/ml (see Fig. S1A in the supplemental material) was exposed to a subinhibitory concentration (31.25 μg/ml) of ciprofloxacin for 1 h. Subsequently, the transcriptomic and proteomic responses were analyzed via transcriptome sequencing (RNA-Seq) and SWATH-MS (sequential window acquisition of all theoretical fragment ion spectra mass spectrometry) (19), respectively. This combined approach provided quantitative expression data for 3,983 out of 3,987 genes in total (20) in transcriptomics and 2,063 proteins in proteomics (21), representing 51.7% of the putative AB5075-UW proteome. The transcriptomics and proteomics data showed that 1,135 genes and 917 proteins were significantly (adjusted *P* value of 0.05) differentially expressed when exposed to 31.25 μg/ml ciprofloxacin (see Data Set S1 in the supplemental material). The Pearson correlation coefficient between mRNA abundance and protein abundance was 0.84 when gene expression and protein abundance data were filtered based on an adjusted *P* value of 0.05 (Fig. 1A).

A total of 153 genes had higher transcript levels, and 159 genes had lower transcript levels, by a log_2_ fold change of >1.5 (adjusted *P* value of 0.05) (Fig. 1B). In contrast, proteomics data showed higher expression of 21 proteins and lower expression of 14 proteins, by a log_2_ fold change of >1.5 (adjusted *P* value of 0.05) (Fig. 1B). These data were filtered to investigate the intersecting genes and proteins. The 16 intersecting abundant genes (and respective proteins) include components of the intact prophage *phiOXA*, components of the CRISPR-Cas system, and the hypothetical proteins ABUW_0369 and ABUW_0098 (Fig. 1C). Induction of prophage gene expression in response to ciprofloxacin stress has been previously described in other pathogenic organisms, such as Salmonella enterica serovar Typhimurium and Staphylococcus aureus, wherein ciprofloxacin is shown to facilitate phage-mediated gene transfer and expression of phage-encoded virulence factors, respectively (22, 23). In contrast, 8 intersecting genes (and representative proteins) showing lower expression include those coding for functions such as hypothetical proteins, the outer membrane protein CarO, and entericidin B (EcnB) (Fig. 1C).

Functional class analysis of differentially expressed genes and proteins identified enrichment of COG (Clusters of Orthologous Genes database) categories associated with DNA replication, recombination and repair, and cell envelope (Fig. 1D). In transcriptomics, several genes associated with DNA damage and repair were highly expressed, including the translesion polymerase genes *umuC* and *umuDAb* (expressed by log_2_ fold changes of 3.0 and 2.6, respectively).

The RND (resistance-nodulation-division) efflux pump AdeIJK has previously been shown to confer intrinsic resistance to ciprofloxacin in the A. baumannii strain ATCC 17978 (17). The genes encoding AdeIJK were not highly expressed in our transcriptomics or proteomics data. However, we conducted MIC tests with AB5075-UW Δ*adeI*, Δ*adeJ*, Δ*adeK*, and Δ*adeN* mutants that have been inactivated by transposon mutation (20) to test their susceptibility to ciprofloxacin (Fig. S1B). AdeN belongs to the TetR family of regulators and represses the activity of AdeIJK efflux pumps (24). The *adeN* mutant had a 2-fold decrease in susceptibility to ciprofloxacin, while *adeIJK* mutants were more susceptible than the parental strain (Fig. S1B). This suggests that while *adeIJK* expression was not induced in AB5075-UW, it plays an important role in intrinsic resistance to ciprofloxacin (25).

### Ciprofloxacin stress increases expression and gene dosage shift near the origin of replication (*oriC*).

As shown in Fig. 1E, when transcriptomics and proteomics data are mapped as a function of gene position in the genome, transcription of genes proximal to the origin of replication (*oriC*) increases. We postulated that this may be due to a higher number of gene copies closer to the *oriC* during ciprofloxacin stress. To show that this was gene dosage dependent, we treated AB5075-UW with ciprofloxacin, using the same conditions as in transcriptomics and proteomics analyses (31.25 μg/ml for 1 h), and performed whole-genome sequencing. As shown in Fig. 1F, the gene dosage closer to *oriC* was much higher in ciprofloxacin-treated cells than in the no-treatment cells. Replication forks in the bacteria start from the *oriC* and extend bidirectionally toward the terminus region of the chromosome. In healthy exponentially growing cells, bidirectional replication of the bacterial chromosome leads to a higher replication-associated gene dosage closer to the *oriC* (26). Genes that are essential for transcription and translation tend to be located close to the *oriC*; as a result, these genes benefit from a higher dosage, and their transcription activity increases, presumably contributing toward bacterial fitness (26). Ciprofloxacin-mediated stalling of replication forks reduces the rate of DNA elongation; however, since new rounds of replication are continuously initiated, the replication-associated *oriC-ter* gene dosage increases, leading to more copies of genes closer to the *oriC*, and thus a higher likelihood of transcription/translation.

### ABUW_0098 is a novel gene that confers tolerance to ciprofloxacin.

The hypothetical gene of unknown function ABUW_0098 and its encoded protein were overexpressed in transcriptomics and proteomics analyses by log_2_ fold changes of 3.26 and 4.10, respectively. To investigate whether ABUW_0098 provides tolerance to ciprofloxacin, we cloned the ABUW_0098 gene with its predicted endogenous promoter (405-bp upstream region of ABUW_0098) into the shuttle vector pVRL1Z (27), and the resulting plasmid, pVRL1Z_ABUW_0098_, was used to complement the AB00272 (ABUW_0098-inactivated) mutant strain (see Fig. S2B in the supplemental material) (20). Growth measurements were performed on five A. baumannii constructs. These included the AB00272 mutant and the AB00272 mutant complemented with pVRL1Z_ABUW_0098_, as well as controls, including the AB5075-UW parental strain, AB5075-UW with empty pVRL1Z plasmid, and the AB00272 mutant with empty pVRL1Z plasmid.

**FIG 2:**
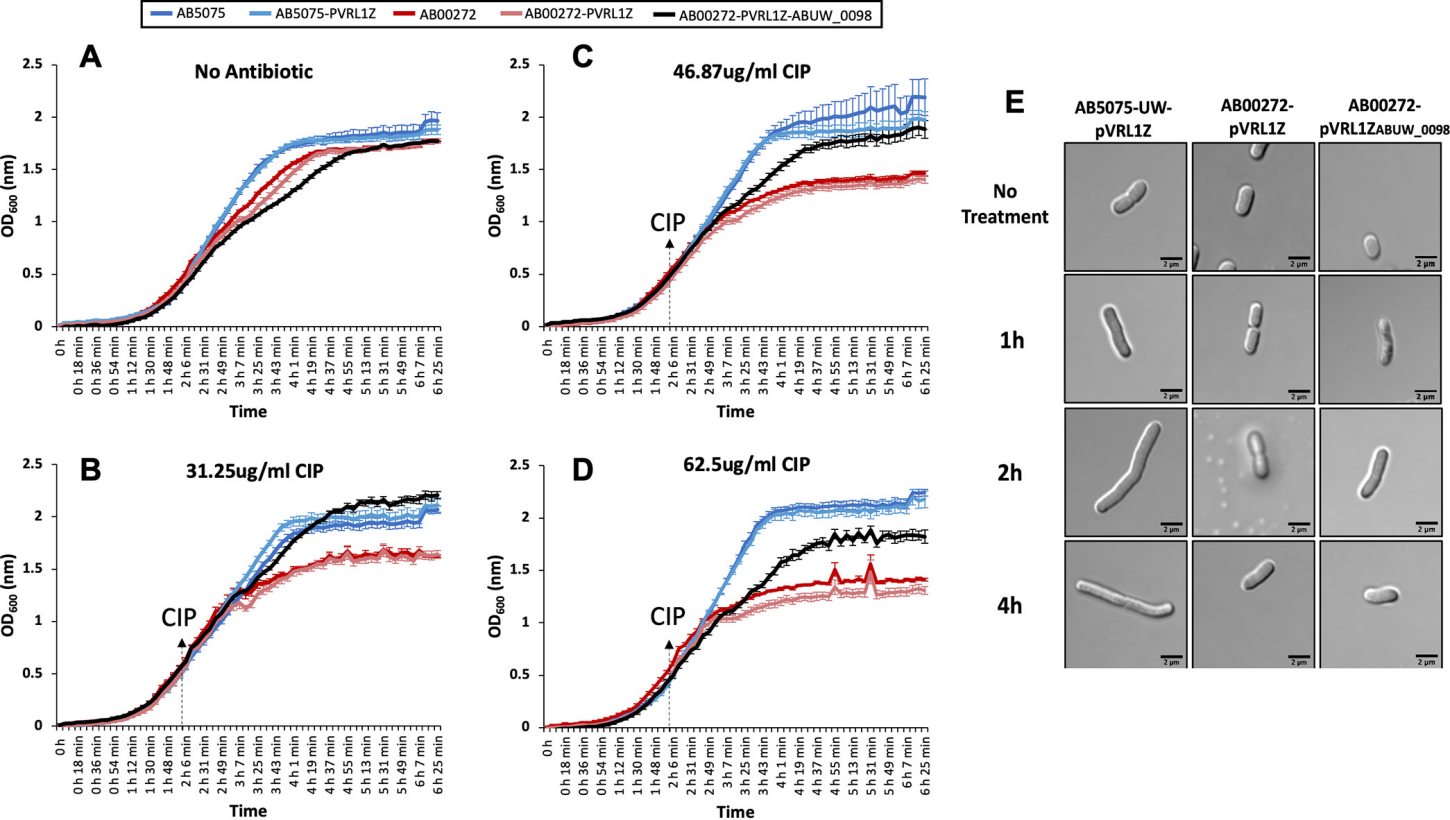
(A) Graph shows 6.25-h growth rate in Mueller-Hinton broth of AB5075-UW (parental strain, blue line), AB5075-UW expressing empty pVRL1Z plasmid (light blue), the AB00272 mutant strain (ABUW_0098 gene insertionally inactivated) (red), AB00272 mutant strain expressing empty pVRL1Z plasmid (light red), and AB00272 mutant strain complemented with pVRL1Z_ABUW_0098_ (black). (B) All AB5075-UW constructs shocked with 31.25 μg/ml of ciprofloxacin after 2 h 6 min of growth. (C) All AB5075-UW constructs shocked with 46.87 μg/ml of ciprofloxacin after 2 h 6 min of growth. (D) All AB5075-UW constructs shocked with 62.5 μg/ml of ciprofloxacin after 2 h 6 min of growth (OD_600_ of ∼0.5). Growth of the ΔABUW_0098 mutant strain, AB00272, was significantly inhibited in the presence of ciprofloxacin. Error bars show the standard errors from three independent experiments. (E) Cell morphology of AB5075-UW expressing empty pVRL1Z plasmid, the AB00272 mutant strain expressing empty pVRL1Z plasmid, and the AB00272 mutant strain complemented with pVRL1Z_ABUW_0098_ grown without antibiotics to mid-log phase (no treatment) followed by exposure to a sub-MIC of ciprofloxacin (31.25 μg/ml) at 1, 2, and 4 h. Scale bar, 2 μm.

We found that AB00272 mutant strain and AB00272 with empty pVRL1Z plasmid showed higher susceptibility to ciprofloxacin than AB5075-UW and AB5075-UW expressing empty pVRL1Z plasmid (Fig. 2A to D). Complementation of the AB00272 mutant with pVRL1Z_ABUW_0098_ restored complete tolerance to ciprofloxacin at 31.25 μg/ml (Fig. 2B) and partial tolerance at 46.87 μg/ml (Fig. 2C) and 62.5 μg/ml (Fig. 2D). Interestingly, in all scenarios changes in the growth rate of all strains were only observed at approximately 45 min (double cell density) after addition of ciprofloxacin.

We sought to investigate the morphologies of all five constructs used in the growth curve experiment (see Fig. S4 in the supplemental material). Cells were imaged at four different time points, which included no treatment (before adding antibiotic) and at 1, 2, and 4 h after addition of 31.25 μg/ml of ciprofloxacin (Fig. 2E; see Fig. S3 in the supplemental material). AB5075-UW and AB5075-UW carrying empty pVRL1Z were elongated by 5 to 10× their original size at the 2-h time point (Fig. S4), consistent with phenotypes observed in E. coli (10, 28). Strikingly, the AB00272 mutant carrying empty pVRL1Z showed no signs of filamentation at any time point (Fig. 2E). Restoring the ABUW_0098 gene in AB00272 did not result in filamentation, similar to AB5075-UW expressing pVRL1Z (Fig. 2E); this could be attributed to the gene copy number. To test this, we introduced pVRL1Z_ABUW_0098_ into AB5075-UW and compared its morphology with that of AB5075-UW expressing empty pVRL1Z at 1, 2, and 4 h after addition of 31.25 μg/ml of ciprofloxacin. We found that while AB5075-UW expressing pVRL1Z_ABUW_0098_ was able to form filamentous structures (see Fig. S6 in the supplemental material), the frequency of filamentation may be less than that of AB5075-UW expressing empty pVRL1Z.

We also investigated whether the downstream gene (ABUW_0099) had any role in filamentation during ciprofloxacin exposure. Growth measurements were performed on two different transposon insertion mutants (AB00274 and AB00275) with this gene inactivated. We found that there was no significant difference in growth rates between the parental AB5075-UW strain and ABUW_0099 mutants when exposed to ciprofloxacin (see Fig. S5 in the supplemental material). Furthermore, unlike the ABUW_0098 mutant, the ability of AB00274 and AB00275 to form filaments was not perturbed in the presence of ciprofloxacin (Fig. S6). A study recently published showed a novel gene, *advA* (ABUW_0096), located approximately 1,700 bp upstream of ABUW_0098, to be localized to cell division sites and to cause cells to adapt filamentous morphologies when deleted (29). Mutations in *advA* caused cells to become hypersensitive to fluoroquinolones and β-lactams.

**FIG 3:**
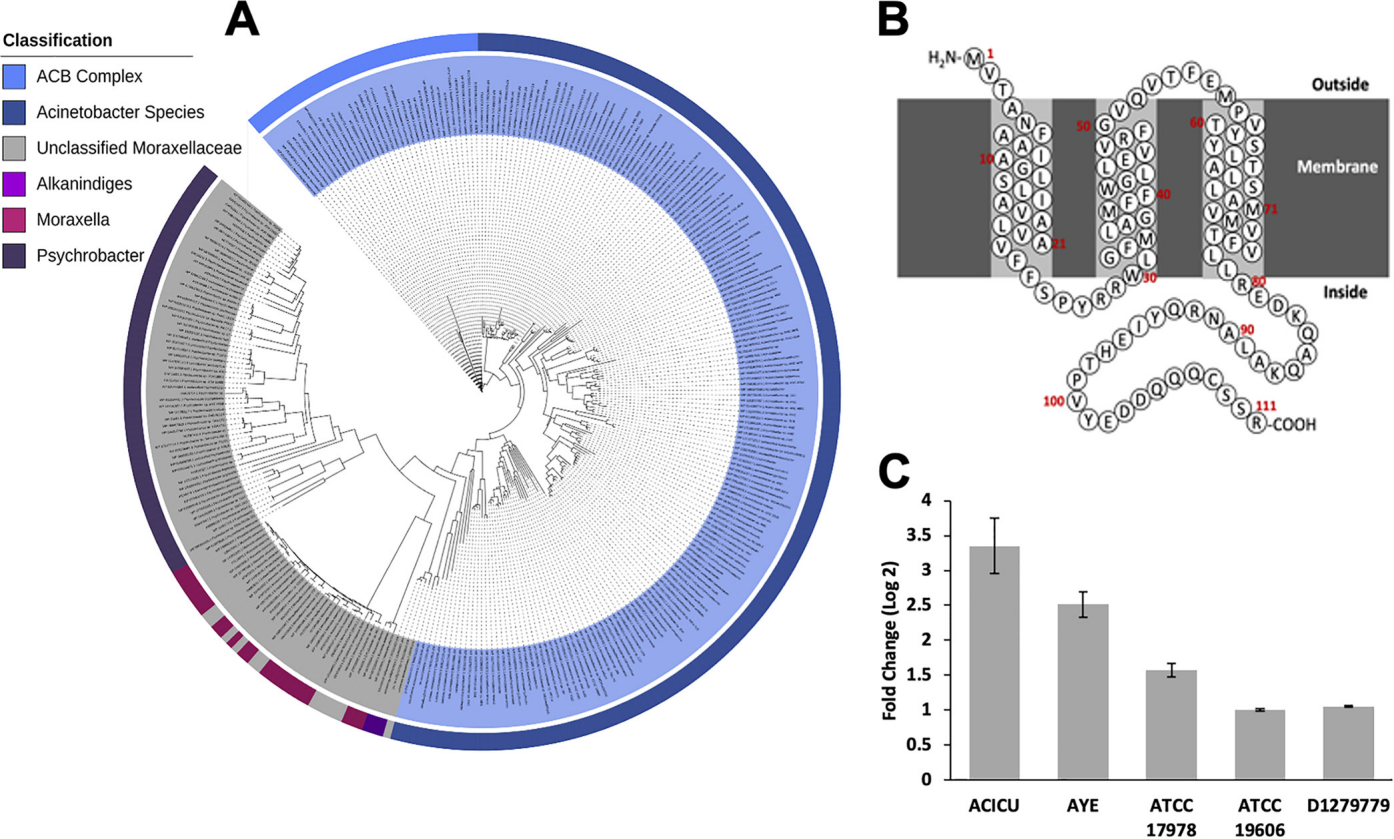
(A) Tree showing the phylogenetic relationship of ABUW_0098 protein. The tree was generated using iTOL from a Clustal Omega alignment of protein sequence obtained from the National Center for Biotechnology Information database. (B) Predicted transmembrane topology of the ABUW_0098 protein (AciT) based on predictions made using TMHMM (36). This protein is predicted to have three transmembrane α-helices. (C) Induction of ABUW_0098 (*aciT*) homologs by ciprofloxacin in A. baumannii strains ACICU, AYE, ATCC 17978, ATCC 19606, and D1279779. A subinhibitory concentration of ciprofloxacin was added to the medium when cells were in the exponential growth phase. The bars represent a change in *aciT* gene expression compared with an untreated control after 1 h of growth in the presence of ciprofloxacin. Error bars show the standard errors from at least 2 biological and 4 technical replicates.

In the well-studied E. coli model, SulA, which is membrane associated (30), is known to inhibit cell division by sequestering FtsZ, preventing the formation of Z rings (31, 32). The inhibition of cell division causes E. coli to become filamentous, while *sulA* mutants are not able to form filaments (31). The ability to form filamentous structures in response to fluroquinolones has been shown to give rise to resistant cells in E. coli (10). Several canonical proteins associated with cell division, including FtsE, FtsX, and SulA, are not found in the core genome of Acinetobacter and *Psychrobacter* species (12). Orthologs of ABUW_0098 are well conserved in Acinetobacter species and are found widely in other bacteria in the family *Moraxellaceae* (Fig. 3A), suggesting the possibility that ABUW_0098 may interfere with FtsZ formation of Z rings. Sequence analysis (33) indicates that the protein encoded by ABUW_0098 has three predicted transmembrane α-helices and is most likely localized to the cell membrane (Fig. 3B). We used quantitative real-time PCR (qRT-PCR) analyses to determine whether ciprofloxacin induces the gene expression of *aciT* orthologs in other A. baumannii strains, including ACICU, AYE, ATCC 17978, ATCC 19606, and D1279779. Each strain was treated with subinhibitory concentrations of ciprofloxacin for 1 h. We found that ciprofloxacin caused a strong induction of *aciT* in ciprofloxacin-resistant A. baumannii strains ACICU and AYE (log_2_ fold change of >2). In contrast, expression of *aciT* in ciprofloxacin-susceptible A. baumannii strain ATCC 17978 was moderately induced (log_2_ fold change of 1.5) and weakly induced in ATCC 19606 (log_2_ fold change of 1) and D1279779 (log_2_ fold change of 1.05) (Fig. 3C). In a recent study with ATCC 17978, the supplementary transcriptomic data presented showed that expression of *aciT* (A1S_3385) was greater than 2-fold at log_2_ when exposed to ciprofloxacin, and introduction of a single *gyrA* resistance allele in ATCC 17978 resulted in ciprofloxacin-induced expression of *aciT* to be greater than 3-fold at log_2_ (17). The variations in ciprofloxacin-induced expression of *aciT* could be attributed to many factors, which include the concentration of antibiotic used, variations at the strain level, or differences in *aciT* induction between ciprofloxacin-susceptible and -resistant strains.

The data presented in this study demonstrate that a previously uncharacterized gene, ABUW_0098, is important for tolerance to ciprofloxacin in A. baumannii. We have named ABUW_0098 the Acinetobacter ciprofloxacin tolerance (*aciT*) gene. In Acinetobacter species, which lack the cell division inhibitor SulA, we propose that AciT may fulfil an equivalent functional role. The discovery of *aciT* demonstrates the significant value of genome-wide expression studies to identify novel drug targets and tolerance factors in multidrug-resistant bacteria.

## MATERIALS AND METHODS

### Bacterial strains, plasmids, reagents, and growth media.

The bacterial strains used in this study include A. baumannii strains AB5075-UW, ATCC 17978, ACICU, and AYE and E. coli strain HST08. AB5075-UW and its transposon mutants were obtained from the Manoil laboratory three-allele collection (20). All bacterial cultures were maintained at 37°C in MH broth with shaking (200 rpm) unless specified otherwise. E. coli strain HST08 was used as a host for plasmid propagation. The parent plasmid used in this study was pVRL1Z (27). Strains carrying pVRL1Z plasmid variants were cultured in low-salt LB medium containing 25 μg/ml Zeocin for E. coli and 300 μg/ml for A. baumannii.

### Gene cloning, PCR, and complementation.

The coding region of ABUW_0098 with its predicted endogenous promoter (450-bp upstream region of ABUW_0098) was purchased from Integrated DNA Technologies (see Table S2 in the supplemental material). This gene fragment was cloned in the pVRL1Z plasmid, using EcoRI and NotI restriction enzymes. Insertion of the gene fragment in the plasmid was confirmed via PCR followed by Sanger sequencing using universal M13 forward and reverse primers (see Table S1 in the supplemental material). Disruption of the ABUW_0098 gene in the mutant strain AB00272 was confirmed by gene-specific primers spanning the ABUW_0098 gene (Table S1).

### Antibiotic susceptibility assays.

Susceptibility assays were conducted in Mueller-Hinton (MH) broth using the both microdilution method as described previously (34).

### Ciprofloxacin growth rate assay.

Overnight cultures of individual AB5075-UW constructs were diluted (1/100) in MH broth and grown in 96-well plates at 37°C with shaking at 200 rpm in the PHERAstar FSX (BMG Labtech, Germany), with optical density (OD) measurements recorded every 6 min. Once the cultures reached an exponential phase of ∼0.5 at the optical density at 600 nm (OD_600_), the appropriate concentration of ciprofloxacin was added, and cultures were allowed to grow continuously for an additional 4 h 20 min with regular OD measurements every 6 min.

### Microscopic imaging.

Overnight cultures of individual strains were diluted (1/100) in MH broth and grown at 37°C with shaking at 200 rpm until cells reached the exponential phase at an OD_600_ of ∼0.5. Approximately 50 μl of cells was harvested at this point and fixed (3% glutaraldehyde–0.1 M phosphate buffer solution). Cultures were then treated with 31.25 μg/ml ciprofloxacin and allowed to grow for a further 4 h. During this time, cells were harvested at 1, 2, and 4 h and fixed immediately. Fixed cells were immobilized on poly-l-lysine-coated cover slides. Imaging was performed with an Olympus FV3000 confocal microscope (Olympus, Tokyo, Japan) using FluoView software (Olympus, Tokyo, Japan). The differential interference contrast (DIC) image was obtained by a 405-nm laser and transmitted detector with a 100× oil immersion objective (Olympus UApo N, 1.49 NA, infinity-corrected 0.13- to 0.19-mm working distance/FN22). Each image was saved in the OIF file format. Image processing was done using ImageJ software.

### Cell treatments.

A. baumannii AB5075-UW cells were grown overnight in 5 ml MH broth. MH broth was reseeded with cultures at 1:100, and the cultures were grown for ∼2 h at 37°C to an OD_600_ of 0.6 with shaking (200 rpm) in 100-ml cultures. Three samples were treated with 31.25 μg/ml ciprofloxacin, whereas the other three samples were not treated and used as the control. Cultures were grown for a further 1 h, at which point they were split into a 30-ml culture for RNA isolation and a 70-ml culture for protein isolation.

### Protein extraction and analysis.

Bacterial cells were prepared for proteomic analysis as detailed in reference 35. For protein extraction, cells were first washed with phosphate-buffered saline (PBS; pH 7.4), resuspended in lysis buffer (50 mM Tris-HCl [pH 8.8], 1% SDS [wt/vol], 8 M urea, EDTA-free protease inhibitor cocktail), and lysed by bead beating. Protein disulfide bonds were reduced using dithiothreitol (DTT) and alkylated using iodoacetamide (IAA). Subsequently, samples were desalted using methanol-chloroform precipitation and redissolved in 50 mM Tris-HCl (pH 8.8)–8 M urea. After the pellet was dissolved, samples were diluted with 50 mM Tris-HCl (pH 8.8) to reduce the concentration of urea to 1.6 M. The bicinchoninic acid (BCA) assay was performed as per manufacturer’s instructions to determine the protein concentrations.

An equal amount of protein sample was digested with LysC protease at 37°C overnight, followed by proteolysis with trypsin at 37°C. The digests were desalted using a C_18_ reverse-phase spin column, and samples were divided into two fractions: one for the generation of the reference ion library, using information-dependent acquisition mass spectrometry (IDA-MS), and another for label-free relative quantification, using SWATH-MS (19).

To create a reference peptide ion library, first, a pool of tryptic peptides was generated by combining a fraction of sample from all the biological conditions. Peptides were prefractionated using strong cation-exchange chromatography and high-pH reverse-phase chromatography. Fractionated peptides were separated by reverse-phase (RP) liquid chromatography using a nano-liquid chromatography (nano-LC) system and detected in a TripleTOF 5600 (SCIEX) mass spectrometer operated in IDA-MS mode. The peptide ion library was generated by searching IDA-MS data against the A. baumannii (strain AB5075-UW) reference protein sequence (3,839 sequences [source, Uniprot]), using the ProteinPilot 5.0 search engine with the Paragon algorithm at a 1% false-discovery rate.

For label-free relative quantification of proteins using SWATH-MS, equal amounts of peptides of the individual samples were separated over a reverse-phase linear gradient of 5.5 to 33% of solvent B (90% [vol/vol] acetonitrile–0.1% [vol/vol] formic acid) over 60 min at a flow rate of 600 nl/min using a nano-LC system in conjunction with a TripleTOF 5600 (SCIEX). The mass spectrometer was operated in positive nanoflow electrospray analysis mode, and SWATH-MS acquisition was performed in a 60-variable *m*/*z* window method over an *m*/*z* range of 400 to 1,250, selected based on the distribution of intensities of the precursor *m*/*z* values in the IDA data sets. Collision energies were calculated for 2+ precursors with *m*/*z* values of the lowest *m*/*z* + 20% for each window width, and a collision energy spread of 5 eV was used.

The reference ion library was imported into PeakView software 2.1 using the SWATH MicroApp 2.0 (SCIEX) and matched against SWATH-MS data from individual replicates. Cumulative protein areas from extracted ion chromatograms, representing the quantitative value of individual proteins, were exported to Excel for further analysis.

### RNA extraction and analysis.

Cells for RNA extraction was centrifuged immediately and suspended in QIAzol (Qiagen). RNA extraction was carried out using the miRNeasy RNA extraction kit from Qiagen, as per the manufacturer’s instructions. DNA was eliminated using the Turbo DNase kit (Ambion) as per the manufacturer’s instructions. RNA integrity was determined using the Agilent Bioanalyzer, followed by rRNA depletion using the Ribo-Zero rRNA removal kit for Gram-negative bacteria (Illumina, Inc., USA). The cDNA library was generated using the TruSeq stranded total RNA sample preparation kit (Illumina, Inc., USA) and samples sequenced on an Illumina HiSeq4000 platform. This yielded 20 million 100-bp paired-end (PE) reads per sample approximately. The EDGE-pro (Estimated Degree of Gene Expression in Prokaryotic Genomes) pipeline was used to align reads to the A. baumannii AB5075-UW reference genome (GenBank accession no. CP008706.1), using Bowtie2, and to generate the raw read counts.

### DNA extraction and analysis.

MH broth was reseeded with A. baumannii AB5075-UW cells at 1:100 from overnight cultures, and the cultures were grown for ∼2 h at 37°C with shaking (200 rpm). Cultures at an OD_600_ of 0.6 with and without the 31.25-μg/ml ciprofloxacin treatment were grown for 1 h at 37°C with shaking. Two biological replicates were prepared. The total DNA of each sample was extracted using the DNeasy UltraClean Microbial kit, following the manufacturer’s instructions. Geneious 2020.2.4 was used to map the raw sequencing reads to the AB5075-UW genome available from the NCBI database (NZ_CP008706.1).

### Quantitative real-time PCR analyses of *aciT* expression.

A. baumannii strains ACICU, AYE, and ATCC 17978 were grown as described above for AB5075-UW RNA and protein extraction. Cultures at an OD_600_ of 0.6 with and without the subinhibitory concentration of ciprofloxacin (15.625 μg/ml for ACICU, 31.25 μg/ml for AYE, and 0.125 μg/ml for ATCC 17978) (Fig. S1C and D) were grown for 1 h at 37°C with shaking. Total RNA was isolated using the miRNeasy RNA extraction kit from Qiagen, as per the manufacturer’s instructions. DNA was eliminated using the Turbo DNase kit (Ambion) as per the manufacturer’s instructions. Reverse transcription and qRT-PCR were performed using the Roche KAPA SYBR Fast one-step qRT-PCR kit, as per manufacturer’s instructions The *rpoB* gene encoding the beta subunit of RNA polymerase was used as an internal reference control.

### Data analysis.

All statistical analysis and data visualization were performed with the R program (v.4.0.0), unless specified otherwise. The DESeq2 package (v.1.28.1), based on the negative binomial generalized linear model with default settings, was used to estimate the differential expression for transcriptomics and proteomics. Differential expression was generated by comparing treated samples against untreated samples. The Benjamini-Hochberg adjustment was used to select differentially expressed genes.

### Data availability.

The DNA and RNA sequence data that support the findings of this study have been deposited in the NCBI Sequence Read Archive (SRA) under accession no. PRJNA673281.
