# Supplementary material for: Identification of a Novel Ciprofloxacin Tolerance Gene, aciT, Which Contributes to Filamentation in Acinetobacter baumannii
Source: Antimicrob Agents Chemother. 2021 May 18;65(6):e01400-20. doi: 10.1128/AAC.01400-20 (PMC8316044; doi:10.1128/AAC.01400-20)
Supplement: Supplementary file 1 [file aac.01400-20-s0001.pdf]

Supplementary material

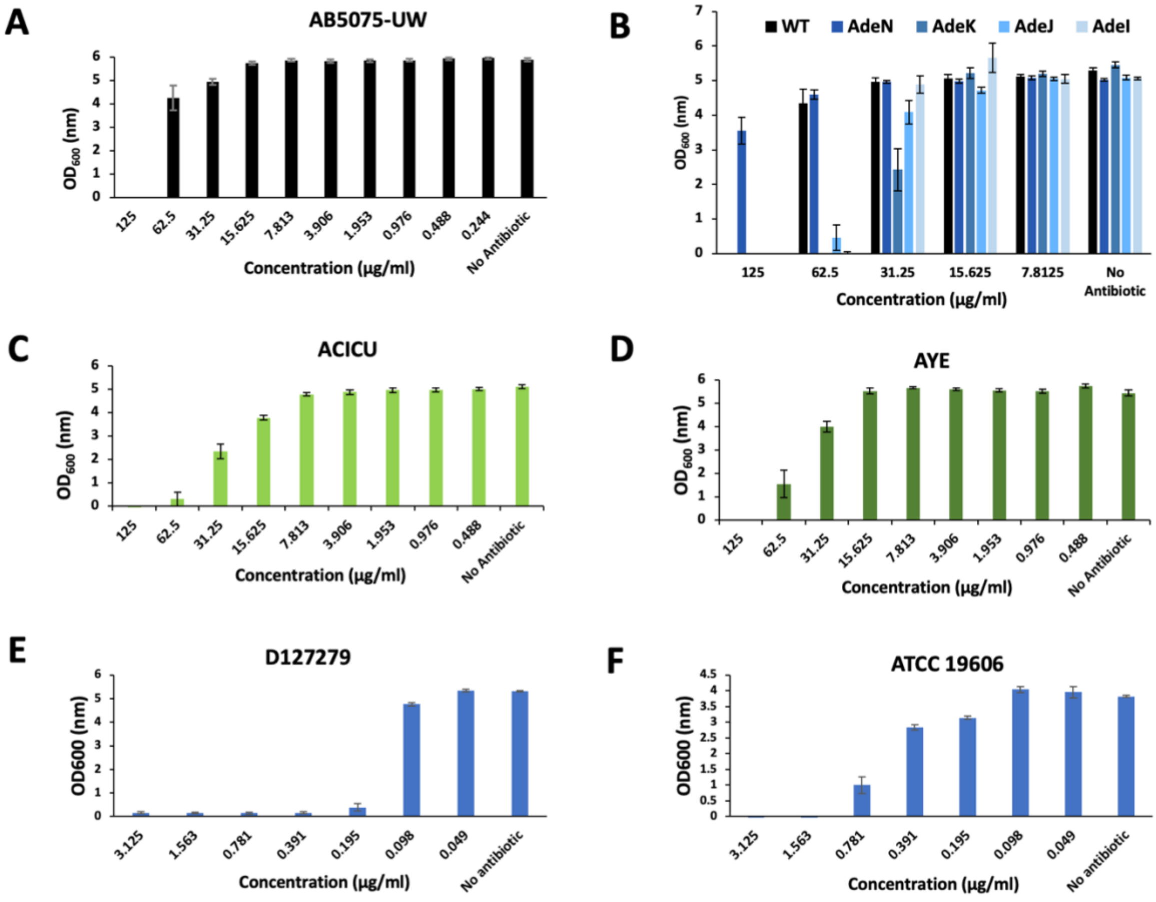

Figure S1.

(A) The susceptibility of AB5075-UW was determined using a minimum inhibitory concentration (MIC) broth microdilution method. The MIC was determined to be 125µg/ml. **(B)** The susceptibility of AB5075-UW (WT), and Tn insertion mutants *ΔadeI* (AB02300), *ΔadeJ* (AB02296), *ΔadeK* (AB02291) and *ΔadeN* (AB04557) were tested using the broth microdilution method. Each strain was subjected to either no ciprofloxacin (growth control - GC) or different concentrations of ciprofloxacin. The optical density was measured after 24h. All mutants (except ΔadeN) were more susceptible to ciprofloxacin when compared WT. Data bars represent geometric mean ± SE (n = 8) of two independent experiments. **(C)** The susceptibility of ACICU was determined using a minimum inhibitory concentration (MIC) broth microdilution method. The MIC was determined to be ~62.5µg/ml. **(D)** The susceptibility of AYE was determined using a minimum inhibitory concentration (MIC) broth microdilution

method. The MIC was determined to be 125µg/ml. (E) The susceptibility of D1279779 was determined using a minimum inhibitory concentration (MIC) broth microdilution method. The MIC was determined to be 0.39µg/ml. (F) The susceptibility of ATCC 19606 was determined using a minimum inhibitory concentration (MIC) broth microdilution method. The MIC was determined to be 1.56µg/ml.

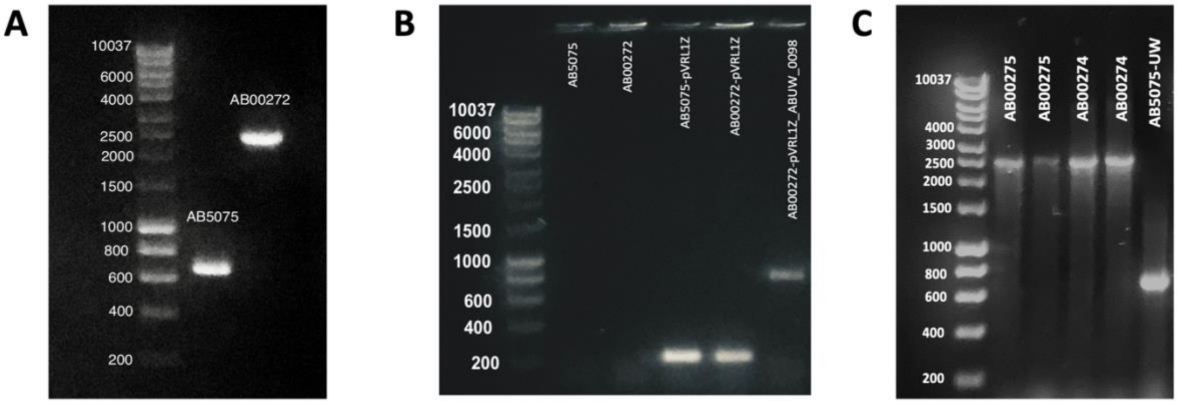

Figure S2.

(A) ABUW_0098 gene-specific primers were used to confirm insertional inactivation of ABUW_0098 gene. The sizes of the molecular weight standards are shown on the left (lane 1). Lane 2 corresponds to AB5075-UW (Wildtype) strain. Lane 3 corresponds to strain AB00272 (ABUW_0098 gene disrupted by the T26 transposon). **(B)** M13 universal primers were used to confirm insertion of ABUW_0098 gene with a 405bp upstream region. The sizes of the molecular weight standards are shown on the left (lane 1). Primers used for the reaction correspond to AB5075-UW (Lane 2), AB00272 (Lane3), AB5075-UW expressing empty vector - pVRL1Z (Lane 4), AB00272 expressing empty vector - pVRL1Z (Lane 5) and AB00272 expressing ABUW_0098 gene with its presumably endogenous promoter (405bp upstream region of ABUW_0098) on a pVRL1Z plasmid (Lane 6). **(C)** ABUW_0099 gene-specific primers were used to confirm insertional inactivation of ABUW_0099 gene. The sizes of the molecular weight standards are shown on the left (lane 1) Lanes 2 and 3 correspond to AB00275; lanes 4 and 5 correspond to AB00274 (ABUW_0099 gene disrupted by the T26 transposon) and lane 6 corresponds to AB5075-UW (Wildtype) strain

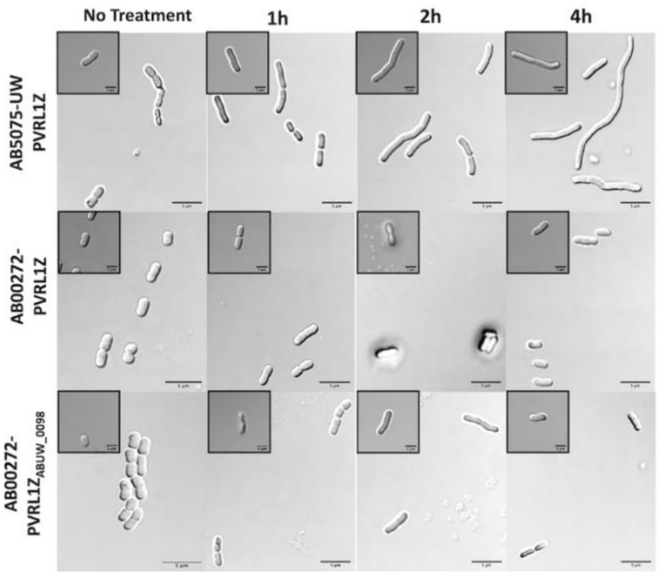

Figure S3.

Cell morphology of AB5075-UW expressing empty pVRL1Z plasmid, AB00272 mutant strain expressing empty pVRL1Z plasmid and AB00272 mutant strain complemented with pVRL1Z$_{ABUW_0098}$ grown without antibiotics to mid-log phase (no treatment) followed by exposure to sub-MIC ciprofloxacin (31.25µg/ml) at 1h, 2h and 4h. Scale bar, 5µm. Inset scale bar, 2µm.

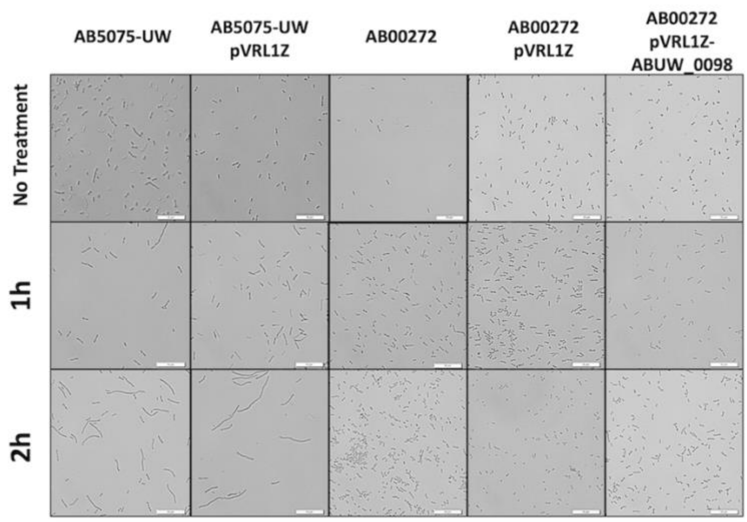

Figure S4.

Cell morphology of AB00272 mutant, AB00272 mutant complemented with $pVRL1Z_{ABUW\_0098}$, as well as controls including AB5075-UW parental strain, AB5075-UW with empty pVRL1Z plasmid and AB00272 mutant with empty pVRL1Z plasmid. Cells were grown to mid-log phase without antibiotics (no treatment) followed by exposure to sub-MIC ciprofloxacin (31.25µg/ml). Cells were harvested for microscopy before adding antibiotics (no treatment) and at 1h and 2h post-treatment. Scale bar, 20µm.

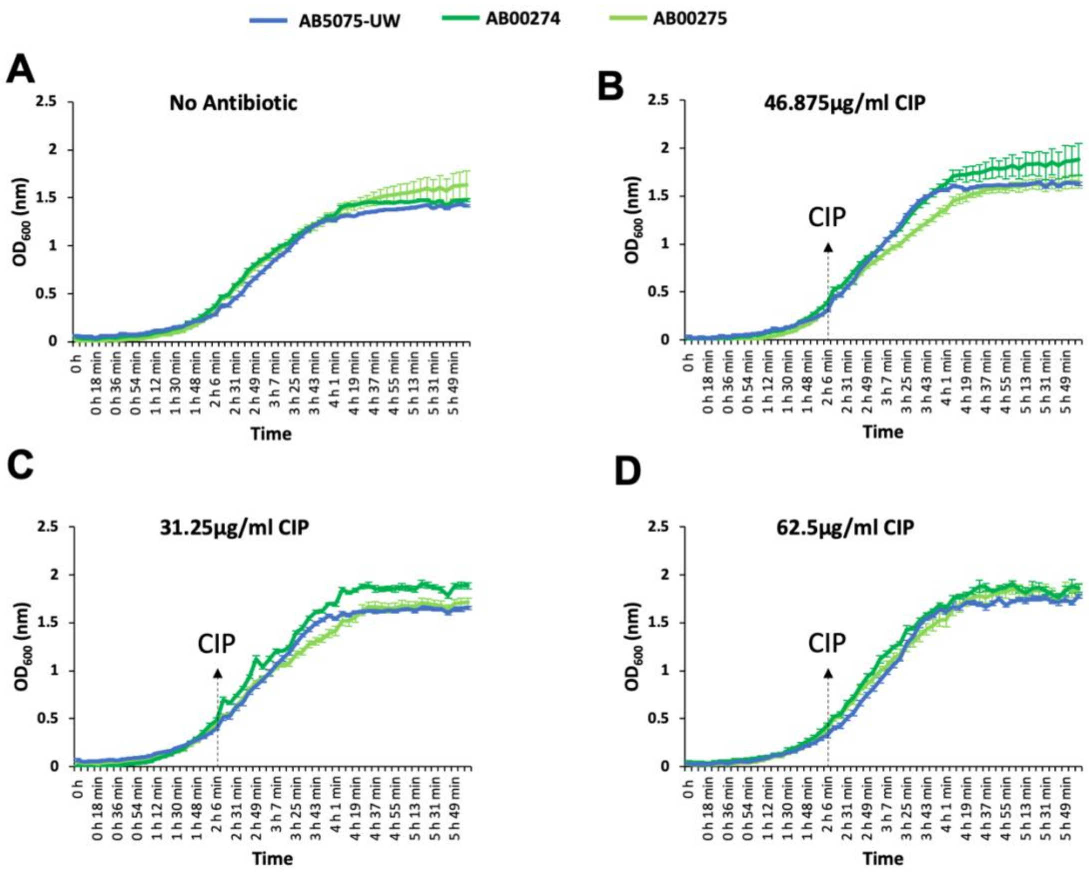

Figure S5.

(**A**) Graph shows 6.25h growth rate in Mueller-Hinton broth of AB5075-UW (parental strain, blue line), AB00274 (green) and AB00275 (light green) mutant strains (ABUW_0099 gene insertionally-inactivated). **(B)** All strains were shocked with 31.25µg/ml of ciprofloxacin after 2h 6min of growth. **(C)** All strains were shocked with 46.87µg/ml of ciprofloxacin after 2h 6min of growth. **(D)** All strains were shocked with 62.5µg/ml of ciprofloxacin after 2h 6min of growth (OD_{600nm} ~0.5). There was no significant difference in the growth of the ΔABUW_0099 mutant strain and AB5075-UW with and without ciprofloxacin. Error bars show the standard error of three biological replicates each.

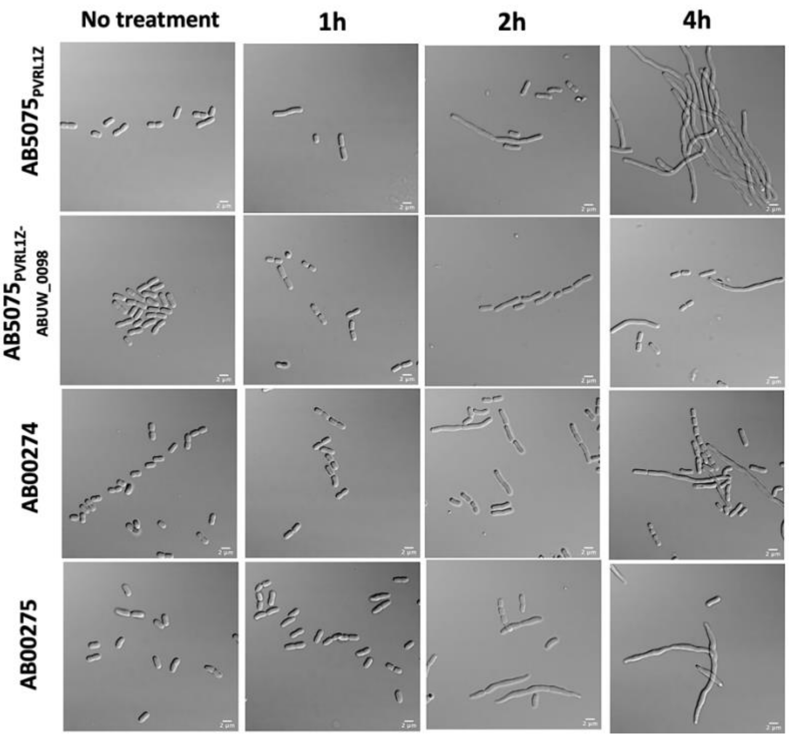

Figure S6.

Cell morphology of AB00274 and AB00275 mutant strains (ABUW_0099 gene insertionally-inactivated) and AB5075-UW complemented with pVRL1Z$_{\text{ABUW_0098}}$, as well as control AB5075-UW with empty pVRL1Z plasmid. Cells were grown to mid-log phase without antibiotics (no treatment) followed by exposure to sub-MIC ciprofloxacin (31.25µg/ml). Cells were harvested for microscopy before adding antibiotics (no treatment) and at 1h, 2h and 4h post-treatment. Scale bar, 2µm.

Table S1.

Primers used in this study.

Primer	Sequence	Description
M13F	TGTAAAACGACGGCCAGT	To confirm insertion of the construct in
M13R	CAGGAAACAGCTATGAC	pVRL1Z plasmid
ABUW_0098F	TCGCATCGACAAATAACA	To confirm the disruption of
ABUW_0098R	TGTTTCGTGCATCGACT	ABUW_0098 gene
ABUW_0099F	CACGAAACAGGCAATGGTGA	To confirm the disruption of
ABUW_0099R	TGCTGCGGACATGAAAGTTC	ABUW_0099 gene
*aciT*_F	CCGTCGTTGGCTTGGTTTTA	To investigate the induction of *aciT* in *A.*
*aciT*_R	TATTGGCGATTTGCCAAAGC	*baumannii* strains via RT-qPCR
*rpoB*_F	CCACGGTTTGCGATTACACA	Housekeeping gene *rpoB* used as a control
*rpoB*_R	CGCATATGGACGTTTCTGCA	in the RT-qPCR experiment.

Table S2.

gBlocks (Intergrated DNA Technologies) Gene fragment used in this study

Description	Sequence
ABUW_0098 gene with a 405bp upstream region	ATAAGCTTGATATCGTGAATAATCATTAAGCGCTGCTCAAGAGTAGCGCTTTTTT<br>TATGCAGAATAGTCATAAAAACAAGCAAGTTATGACAAAATTATGCACAGAAAAA<br>AGGCAATAAAAAAAACTGAAAAAAACTTATTTTGAGGCAAAAAAACAGCATAGTT<br>TTTTGTAATGACACGCCAGAATTGATGCCATATAGGAAGTAGAACTTTTTTAAAT<br>TTACAAAAATGTAATGACAAAAGAATGTTGCGACAAAGTATGTCGCATCGACAAA<br>TAACAGGTCTTTTTTTCTAAAAAAAAACATCCATAATGCTTTCTAAGCTTCAACA<br>CAAATCATGAATTAAAGTTGTTTTTGGAATGAGGTCTGGTGTTCAGACCGAATGT<br>TTTTAGACGTTTTCATTAAGACGGAGGTTTCTATGGTAACAGCGAATTTTGCCGC<br>TATCGCCGGACTCAGCTTAATTGCTGTCGCGCTTGTTGCTGTGTTCTTTTCTCCT<br>TACCGTCGTTGGCTTGGTTTTATGCTTGCAGGGATGTTTTTTTGGGGGCTATTGG<br>AAGTGGTCCGTTTTGGAGTTCAAGTCACTTTTGAAATGCCAGTCACATATAGTTA<br>TTTAACTGCACTAAGTCTAGCGATGGTAATGGTTACATTTGTTTTATTACGTGAG<br>GATAAACAGGCACAGAAGGCTTTGGCAAATCGCCAATATATAGAACACACGCCAG<br>TATATGAAGATGATCAGCAGCAATGTTCTAGCCGATAACGGCCGCCACCGCGG

Data S1.

Transcriptomics and Proteomics fold-change data
